# Functional Coverage of the Human Genome by Existing Structures, Structural Genomics Targets, and Homology Models

**DOI:** 10.1371/journal.pcbi.0010031

**Published:** 2005-08-19

**Authors:** Lei Xie, Philip E Bourne

**Affiliations:** San Diego Supercomputer Center and Department of Pharmacology, University of California, San Diego, California, United States of America; European Bioinformatics Institute, United Kingdom

## Abstract

The bias in protein structure and function space resulting from experimental limitations and targeting of particular functional classes of proteins by structural biologists has long been recognized, but never continuously quantified. Using the Enzyme Commission and the Gene Ontology classifications as a reference frame, and integrating structure data from the Protein Data Bank (PDB), target sequences from the structural genomics projects, structure homology derived from the SUPERFAMILY database, and genome annotations from Ensembl and NCBI, we provide a quantified view, both at the domain and whole-protein levels, of the current and projected coverage of protein structure and function space relative to the human genome. Protein structures currently provide at least one domain that covers 37% of the functional classes identified in the genome; whole structure coverage exists for 25% of the genome. If all the structural genomics targets were solved (twice the current number of structures in the PDB), it is estimated that structures of one domain would cover 69% of the functional classes identified and complete structure coverage would be 44%. Homology models from existing experimental structures extend the 37% coverage to 56% of the genome as single domains and 25% to 31% for complete structures. Coverage from homology models is not evenly distributed by protein family, reflecting differing degrees of sequence and structure divergence within families. While these data provide coverage, conversely, they also systematically highlight functional classes of proteins for which structures should be determined. Current key functional families without structure representation are highlighted here; updated information on the “most wanted list” that should be solved is available on a weekly basis from http://function.rcsb.org:8080/pdb/function_distribution/index.html.

## Synopsis

The sequencing of the human genome provides biologists with new opportunities to understand the molecular basis of physiological processes and disease states. To take full advantage of these opportunities, the three-dimensional structures of the gene products are needed to provide the appropriate level of detail. Since protein structure determination lags behind protein sequence determination, an important and ongoing question becomes: what degree of coverage of the human proteome do we have from experimental structures, and what can we infer by modeling? Or, turning the question around: what structures do we need to determine (the “most wanted list”) to further our understanding of the human condition? This paper addresses these questions through integration of existing data resources correlated using comparative functional features, namely the Gene Ontology, which describes biochemical process, molecular function, and cellular location for all types of proteins, and the Enzyme Commission classification for enzymes. Genetic disease states are linked through the Online Mendelian Inheritance in Man resource. Readers can ask their own questions of the resource at http://function.rcsb.org:8080/pdb/function_distribution/index.html. The resource should prove particularly useful to the structural genomics community as it strives to undertake large-scale structure determination with a goal of improving the understanding of protein functional space.

## Introduction

The three-dimensional structure of a protein is an essential component in elucidating the biological function(s) at the molecular level and in understanding the details of molecular recognition. Traditional structural biology supports a paradigm in which biochemical evidence of function is confirmed and further understood through the study of structure [1]. Structural genomics [2] has changed this paradigm, being motivated by a variety of criteria, including a desire to increase the coverage of known fold space [3]. Concomitantly, complete genome sequences are becoming available at an increasing rate and both putative functions and structures defined for coding regions. Even given the limitations of these assignments, it is an appropriate time to assess the current coverage of protein structure space from a functional perspective relative to the perceived functional coverage of complete genomes, notably human. Further, the registering of structural genomics targets (sequences subject to structure determination) by most projects worldwide [4] provides an excellent opportunity to assess what the perceived coverage of functional space by structure will likely be going forward. This paper makes this assessment, discusses where a change of strategy in selecting targets may be appropriate, and reports on functional classes that are well represented in the human genome but without the existence of structures—the so-called “most wanted list.”

Many authors have noted the structural and functional bias in the Protein Data Bank (PDB), but few have attempted to quantify it [5–8]. Rather, general statements are made that refer to the limitations associated with structure determination methods, such as the propensity for small, globular, soluble proteins solved by X-ray crystallography and nuclear magnetic resonance. Beyond physical limitations, there is a bias toward proteins identified as potential drug targets and a historical bias toward structures that, without the benefit of modern techniques, were, from the point of view of protein isolation and structure determination, the most tractable. Where does that put us today, and how can we estimate this bias? A problem that has thwarted such studies is the lack of a common reference frame. This problem has been partially addressed by systems of consistent nomenclature; notwithstanding, depth of coverage is neither complete nor consistent across protein families. Recently, the Enzyme Commission (EC) classification has been used to study the relationships among sequence, structure, and functions [9–15]. Similarly, the Gene Ontology (GO) [16], while still evolving, provides a consistent view of molecular function, biological process, and cell component beyond enzymes. Further, with consistent sequence annotation as a common feature between resources describing structure and human genetic disease, structure–disease relationships can be inferred. Inference requires that care must be taken to assess the statistical significance of the outcome. Using EC and GO to define a common functional framework and highly significant sequence relationships to infer relationships between structure (either solved or under study) and disease, we can measure the biased nature of the PDB and the structures under consideration by structural genomics and suggest protein structures that should be determined to further our understanding of structure and function space.

## Results/Discussion

The relationship between protein structures and their function(s) is complex. A single structure superfamily often displays variations in function. Conversely, the same function can be achieved by proteins with different structures [10,11]. Domain recombination and shuffling leads to further functional diversity [17–19]; hence, this study is undertaken at the level of both single domain and whole protein to provide an in-depth view of the functional distribution of protein structures. Single-domain coverage is defined such that at least one domain in the protein has structural information available from the PDB, structural genomics, or homology models. Whole-protein coverage means that structure information for all domains, including their organization, can be directly or indirectly inferred. Similarities between functional distributions from the human genome and from experimental structures or theoretical models are measured with Kendall's tau correlation, which ranges from −1.0 to 1.0. A large positive value indicates that two measurements have similar ranks. The structure–function relationship analysis is based on non-redundant sequence clusters with less than 40% sequence identity and 90% overlap, since functional similarity usually breaks down below these thresholds [10,15].

### The Functional Bias of PDB Structures

As stated, several studies have noted structural and functional bias in the PDB [5–8]. In general, protein domains such as transmembrane domains, low complexity regions, and disordered regions, which are not suited to current structure determination methods by X-ray crystallography and nuclear magnetic resonance, are highly underrepresented in the PDB. The columns labeled “PDB/Genome” in Tables 1–4 quantify this bias relative to the known functional classification within the human genome using EC (Table 1) and GO (Tables 2–4) classifications. This bias is examined from the perspective of both a single domain and the whole structure, since many proteins have intracellular and extracellular domains that have been solved without their domain spanning regions. For example, proteins associated with transporter activity (Table 2) have the lowest coverage at the domain level (21.0%), but are further underrepresented at the structure level (12.1%) because of the presence of transmembrane domains. Proteins with two or more contiguous domains, where each of the domains has structure information available, may result in different structures when those domains are swapped. This impacts the observed relationship between values of coverage and correlation computed with Kendall's tau (see Materials and Methods) for single domains versus whole structures, as will be described subsequently.

**Table 1:**
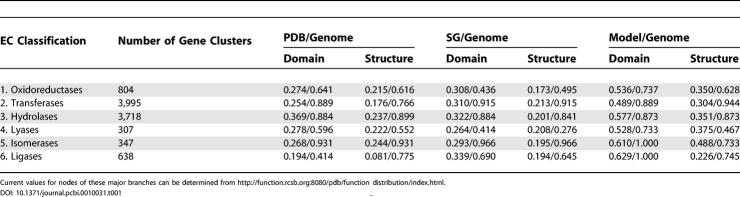
Coverage/Kendall's Tau Correlations for Major Categories of Enzyme for Both Single Domains and Whole Proteins

Current values for nodes of these major branches can be determined from http://function.rcsb.org:8080/pdb/function_distribution/index.html.

**Table 2:**
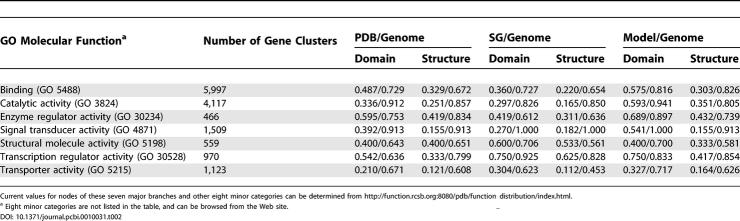
Coverage/Kendall's Tau Correlations for Major Categories of GO Molecular Function for Both Single Domains and Whole Proteins

Current values for nodes of these seven major branches and other eight minor categories can be determined from http://function.rcsb.org:8080/pdb/function_distribution/index.html.

^a^ Eight minor categories are not listed in the table, and can be browsed from the Web site.

Beyond obvious experimental limitations, skewed functional distributions of PDB structures are observed for almost all types and levels of EC and GO classifications (Tables 1–4). For example, consider classification by EC number at all levels (only the top-level EC classification is given in Table 1, but current values for all levels of the EC hierarchy are available from the Web site). The correlation coefficients by Kendall's tau between the genome sequences and PDB structures with single-domain coverage for EC: *.*.*.* (all), 2.*.*.* (transferases), EC 2.7.*.* (transferring phosphorous-containing groups), and EC 2.7.1.* (phosphotransferase with an alcohol group as acceptor) are 0.867, 0.889, 0.806, and 0.383, with coverage of 29.9%, 25.4%, 28.7%, and 32.1%, respectively. Thus, even for one of the most structurally studied superfamilies, the protein kinase-like superfamily (all belong to EC 2.7.1), the structures of the majority of atypical kinases (proteins that phosphorylate a variety of substrates) have not been determined, and the protein kinase family itself is slightly underrepresented. The Kendall's tau correlation coefficient is only 0.383 and 0.192 for single-domain and whole-protein coverage, respectively, for the protein kinase-like superfamily.

The functional bias of PDB structures is also notable when using GO molecular function annotations that extend beyond enzyme activity. A total of 16,211 proteins within the human genome can be annotated at this time. As shown in Table 2, according to their Kendall's tau at the whole structure level, several subcategories are underrepresented, notably transporter activity (already noted) and structural molecule activity. Looking deeper (refer to http://function.rcsb.org:8080/pdb/function_distribution/index.html), there are 16 GO subcategories of molecular function associated with structural molecule activity. Twelve of them have been mapped to the human genome. The most structurally underrepresented proteins include the structural constituent of ribosome (two structures but 180 annotations), of myelin sheath (zero structures and two annotations), of epidermis (zero structures and six annotations), of tooth enamel (zero structures and five annotations), of bone (zero structures and three annotations), of chorion (zero structures and one annotation), and of cell wall (zero structures and one annotation).

Table 3 provides the distribution of protein domains and whole structures according to the subcategories of GO biological process. A total of 14,876 proteins within the human genome can be annotated at this time. Thus, biological process is less well characterized than molecular function, presumably since molecular function cannot necessarily be related to a role in a complex biological process. Notwithstanding, both single-domain and whole-protein structures with an identified role in cellular process are underrepresented.

**Table 3:**
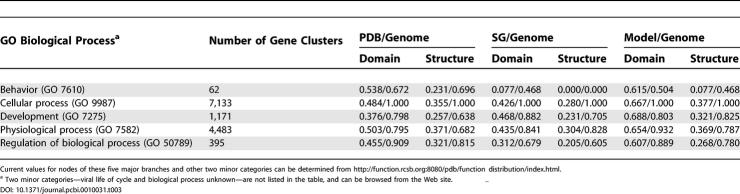
Coverage/Kendall's Tau Correlations for Major Categories of GO Biological Process for Both Single Domains and Whole Proteins

Current values for nodes of these five major branches and other two minor categories can be determined from http://function.rcsb.org:8080/pdb/function_distribution/index.html.

^a^ Two minor categories—viral life of cycle and biological process unknown—are not listed in the table, and can be browsed from the Web site.

**Table 4:**
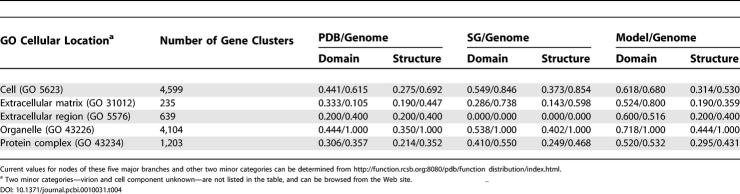
Coverage/Kendall's Tau Correlations for Major Categories of GO Cell Component for Both Single Domains and Whole Proteins

Current values for nodes of these five major branches and other two minor categories can be determined from http://function.rcsb.org:8080/pdb/function_distribution/index.html.

^a^ Two minor categories—virion and cell component unknown—are not listed in the table, and can be browsed from the Web site.

Table 4 provides the distribution of protein domains according to the subcategories of GO cell component. Overall, the distribution between PDB structures and the human genome is comparable, with a Kendall's tau of 0.714; however, proteins identified within the cell (GO 5623) are underrepresented at both the structure and domain levels. Coverage is not as favorable as distribution: only 38.3% of the subcategories of cell location have at least one structure domain representative. Of those, the vast majority are annotated as cell (4,599 out of 8,936 gene clusters). Under cell (see http://function.rcsb.org:8080/pdb/function_distribution/index.html), there are 12 subcategories that have been assigned to the human genome, five have no structure representation and seven have at least one structure domain representative (membrane [3,354 gene and 236 structure clusters], intracellular [1,237 gene and 169 structure clusters], cell surface [23 gene and four structure clusters], cell projection [26 gene and one structure cluster], cell fraction [621 gene and 75 structure clusters], apical part of cell [six gene and one structure cluster], and basal part of cell (two gene and one structure cluster]). As expected, membrane is structurally underrepresented, and intracellular and cell fraction is structurally overrepresented. There is no structural information available for five small subcategories of cell: site of polarized growth (three gene clusters), periplasmic space (three gene clusters), midbody (one gene cluster), external encapsulation (two gene clusters), and cell soma (one gene cluster).

### Structural Coverage of Human Genetic Diseases

Three-dimensional protein structures are important in understanding the mechanisms of human genetic diseases [20], predicting the effect of non-synonymous single nucleotide polymorphisms [20,21], and developing new personalized medicines [22]. For example, a recent study highlights the application of PDB structure and homology models in understanding a predisposition to breast and ovarian cancer [23]. However, the current identification and coverage of human genetic disease space, as identified by the Online Mendelian Inheritance in Man (OMIM), is limited: 218 non-redundant human genome sequence clusters, 46 structure clusters, and 34 structural genomics target clusters. The PDB currently covers 69.9% of the disease categories described by OMIM, but the distribution based on class of disease is uneven. For example, diseases of the central nervous system have the largest single representation (20 structure clusters) with a disproportionately large number of structural genomics targets (23 target clusters). Blood and lymph based diseases have a disproportionate number of ten solved structures, but an underrepresentation of targets (three clusters). Conversely, diseases of the ear, nose, and throat are underrepresented (six structure and seven target clusters). Overall, cancers have an appropriate level of structures and targets; however, digging deeper reveals a different situation. For example, there are no structures available for the five human proteins that have been associated with prostate cancer, although homology models can be inferred for domains such as prostate specific kallikrein of serine proteases [24]. Data showing measurable differences in protein and gene regulatory networks between the early- and the late-stage prostate cancer [25] only highlight the need to further understand the structural basis of this disease. Human genetic disease distributions, while limited, are undoubtedly influenced by historical precedent, preventative and treatable conditions, and social and, hence, funding pressures.

### The Contributions of Homology Modeling

While the number of three-dimensional structures of proteins has increased close to the near-exponential rate predicted by Dickerson in 1978 as number of structures = exp(0.19 × year) [26], there are still a vast number of protein sequences without structure information available. Homology modeling can potentially provide putative structure information for these sequences to facilitate our understanding of their function and evolution [27,28]. Reliable homology modeling usually requires that the query sequence share at least 30% sequence identity with the template structure for each domain [27]. Domain rearrangements and lack of domain structures reduce the effectiveness of homology modeling for whole structures, as shown in the columns labeled “Model/Genome” in Tables 1–4. In almost all EC and GO classifications, coverage and distribution falls for whole structures versus domains. From a biological perspective, modeling of only a subset of domains within a structure limits the value of modeling.

As expected, the distribution of homology models is highly correlated with the availability of PDB structures. Single-domain coverage across the whole human genome indicates that our ability to provide homology models for domains in the different GO molecular function categories varies from 32% to 75%. For the modeling of whole proteins, coverage drops, varying from 16% to 41%. Transporter activity and signal transducer activity is the most difficult to model at the whole-protein level. GO functional coverage for signal transducer activity drops from 0.541 to 0.155. Thus, while catalytic domains involved in signal transduction are well represented and can be modeled in 54% of cases, these data quantitatively show that the associated non-transmembrane domains of the whole protein are significantly underrepresented, thereby limiting our ability to model whole proteins in 38% of cases.

Considering enzymes alone, our ability to homology model single domains is fairly evenly distributed across all major classes (Table 1). At the whole-protein level, this picture changes. Retaining a high Kendall's tau even as coverage drops significantly could imply that functional diversity comes primarily from domain recombination rather than from new domains that cannot be modeled. Indeed, it has recently been reported that contemporary ligases evolved by domain fusions [29], a fact supported by a relatively small drop in Kendall's tau from 1.000 to 0.745 for single domains versus the whole protein.

Low correlation within a functional class implies that homology models can be inferred from structures in different functional subclasses and other species. For example, in the oxidoreductases (EC 1.x.x.x ), five classifications (1.7, acting on other nitrogenous compounds as donors; 1.9, acting on a heme group of donors; 1.10, acting on diphenols and related substances as donors; 1.17, acting on –CH_2_ groups; and 1.97, other oxidoreductase) are not structurally covered at all. However, with the exception of 1.97, other oxidoreductase, the proteins in the four remaining subclasses can be modeled from structural templates present in the other functional subcategories, implying a close evolutionary relationship within this functional class.

Conversely, experimental structural coverage is more critical for functional classes with more distinct evolutional origins, such as the protein kinase-like superfamily, which is in the transferase category. It has been suggested [30] that atypical kinases diverged early in evolution from protein kinases; therefore, homology models of atypical kinases derived from protein kinases are likely insufficient to infer their functional and evolutional roles. In 13% of cases, while the homology model is identified to belong to the protein kinase-like superfamily, the specific family cannot be determined.

### The Contribution of Structural Genomics

One approach to the selection of structural genomics targets has been to focus on increasing the coverage of fold space [31–33]. A recent review suggests that the first phase of structural genomics has been successful in this regard [34]. It is anticipated that functional roles will be given greater precedence in future phases of the project [35–37]. If so, a question to address is: does the current complement of structural genomics targets and the structures solved by these projects reduce the functional bias present in the PDB? The short answer is yes, but only significantly in some functional categories (Tables 1–4, columns labeled “SG/Genome”) and assuming two to three times the number of structures than we have now (based on the relative numbers of clusters between structure genomics targets and PDB structures, given a 40% sequence identity cutoff).

Within the enzymes (Table 1), ligases will benefit the most and lyases the least. Based on GO molecular function (Table 2), structural molecule activity and transcription regulator activity (single domain) will be impacted the most; binding, catalytic activity, signal transducer activity, and transporter activity the least. In terms of GO biological processes (Table 3), structural genomics will contribute almost nothing to our understanding of behavior and about equally to cellular processes, development, physiological processes, and regulation of biological processes. Finally, current structural genomics targets will not contribute to our understanding of extracellular region of cell component (Table 4). The most notable impact of structural genomics overall is in our potential understanding of transcription regulator activity, which shows an improvement in coverage from 0.542 to 0.750 and an improved Kendall's tau of 0.636 to 0.925 for a single domain.

Drilling down into one of these categories, the previously described structurally underrepresented GO class for molecular function—namely, structural molecule—becomes better populated such that targets will increase the coverage of the structural constituent of tooth enamel (one structure but five annotations), of myelin sheath (one structure and two annotations), and of ribosome (48 structures and 180 annotations). There remains no anticipated experimental structure information for the structural constituent of epidermis, bone, chorion, and cell wall (total 11 annotations).

Given these findings, it is timely to consider the choice of structural genomics targets. It has been suggested that solving the structures of proteins from the 5,000 Pfam families will cover more of fold space than focusing on a single genome [38]. Here, we look at target selection from a functional perspective and provide a tool for comparing the functional coverage by the existing PDB and what the existing complement of structural genomics targets do to that functional coverage. The remainder of the paper considers one application of the tool in providing a strategy for selecting structures that could be used to maximize our understanding of structure–function relationships with respect to the human genome.

### Defining Structures That Should Be Determined

To date, approximately 50% of human genes (16,211 terms for GO molecular function, 14,876 terms for GO biological process, and 13,322 terms for GO cell component) have at least one GO annotation. However, approximately 70% of these GO molecular function categories are yet to be covered by experimental structures with even one identifiable domain. The structural coverage of the human genome is even lower with respect to sequence space: approximately 10% coverage by structure at 40% sequence identity. Stated another way, 5% of the human genome, which covers 30% of functional space, has structure representation for at least one domain in a protein. If all current structural targets were determined, it is estimated that coverage of the human genome and functional space would increase to 20% and 50%, respectively. Homology modeling would increase genome and functional coverage to 40% and 60%, but what these putative high-throughput models add to our understanding of molecular function remains questionable. When taking domain recombination into account, the functional coverage of the human genome by existing experimental structures and anticipated structures being determined by structure genomics decreases to approximately 25% of the functional space.

This lack of coverage perhaps calls for a new strategy to select targets for structure determination. Here, one such strategy is outlined for choosing targets to increase the coverage of functional space. It is based on the following criteria: (1) functional categories are preferred where proteins with experimental or theoretical three-dimensional models are underrepresented; (2) from (1), proteins without SCOP superfamily assignments are preferred; (3) if the protein is identified as being associated with a disease or is identified in multiple functional categories, it has a higher priority; and (4) less experimentally tractable proteins—for example, those with transmembrane segments—can be filtered out.

From our initial analysis, approximately 2,000 non-redundant human genes with GO annotation have no experimental structure in the PDB, nor are they identified structural genomics targets or amenable to homology modeling. Of this 2,000, approximately 50% include transmembrane domains. After removing transmembrane and low-complex regions, about 1,800 include at least one domain that is potentially solvable. The most understudied proteins of this 1,800 are various types of transporters and receptors. It should be noted that it requires fewer targets to cover this functional space than the equivalent sequence space.

Ranked by the size of the cluster of proteins, examples of the most pressing biological molecule functions for which structural representation is needed and soluble structure domains are probably present are listed here (Tables 5 and 6) and at http://function.rcsb.org:8080/pdb/function_distribution/index.html, which is updated regularly. For catalytic activity, most of them are involved in protein syntheses and gene regulation. For binding, most of them are involved in signal transduction and have additional benefit as potential drug targets.

**Table 5:**
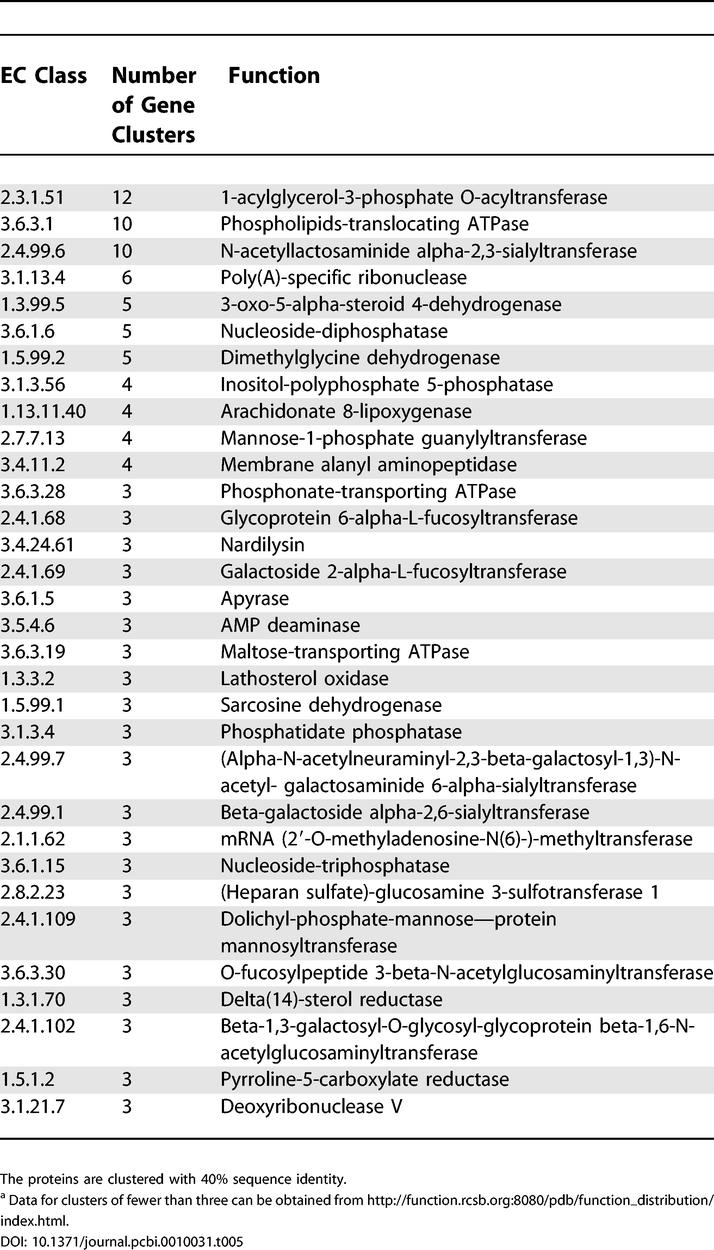
Most Wanted Structures According to EC Numbers^a^

The proteins are clustered with 40% sequence identity.

^a^ Data for clusters of fewer than three can be obtained from http://function.rcsb.org:8080/pdb/function_distribution/index.html.

**Table 6:**
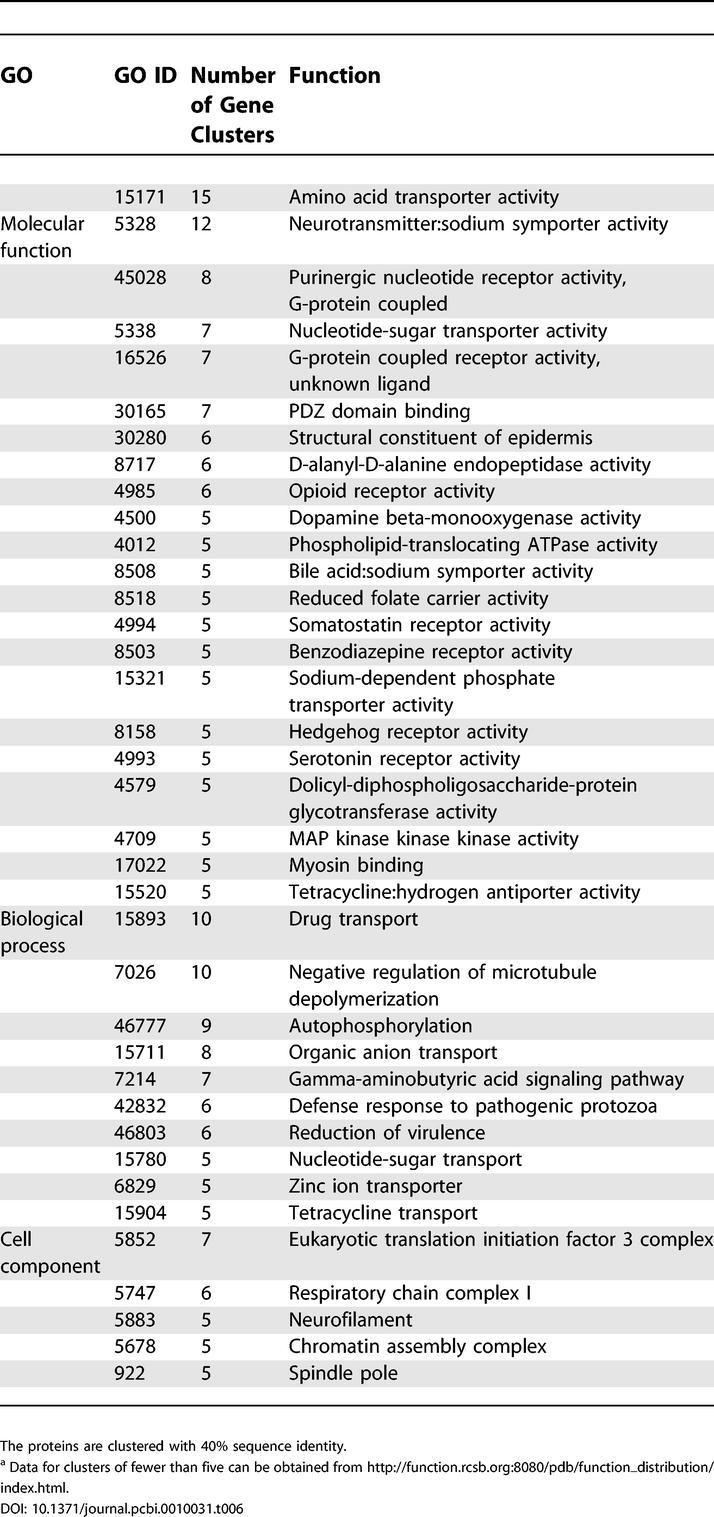
Most Wanted Structures According to GO Classification^a^

The proteins are clustered with 40% sequence identity.

^a^ Data for clusters of fewer than five can be obtained from http://function.rcsb.org:8080/pdb/function_distribution/index.html.

Several genes without experimental structures and not found in the structural genomics target list are annotated by both GO and disease terms (see http://function.rcsb.org:8080/pdb/function_distribution/index.html). For example, congenital adrenal hyperplasia is associated with three gene clusters. Two of them are annotated with oxygen binding (GO ID: 19825), and one with steroid 11-beta-monooxygenase activity (GO ID: 4507).

In summary, by using common annotation as found in the GO and the EC classification scheme, we have been able to correlate the biological functions of proteins and their constituent domains for both experimentally derived structures and those under determination by structural genomics projects worldwide. Further, by using empirical sequence limitations known from homology modeling experiments and by clustering human genome sequences according to sequence identity, we can estimate the impact that current structure determination strategies will have on our understanding of structure–function relationships from homology modeling. Finally, by introducing relationships between gene products and known disease states, we have provided pointers for choosing structures to be determined to have the maximum impact on our understanding of human genetic disease. To facilitate these choices, a Web resource has been established at http://function.rcsb.org:8080/pdb/function_distribution/index.html to allow readers to make their own assessments of the progress of structural biology. The resource will be updated on a weekly basis to provide a current view. The resource itself will be the subject of a separate publication.

## Materials and Methods

### Date sources and annotation mapping.

The human genome sequences (version 26.35.1) were downloaded from Ensembl database [39]. Wild-type sequences associated with PDB structures were generated by associating the structural sequence with that from UniProt [40] using database cross references records. Subsequently, all wild-type PDB sequences of the human proteins were mapped to the genes in the human genome through sequence alignment using Blast [41]. A gene was considered to have a structure representation if it had 100% sequence identity with the wild-type sequence of the PDB structure. Structural genomics targets were taken from targetdb [42], the worldwide repository of all sequences representing structures being attempted. Among more than 5,000 registered human target sequences, there were 3,141 and 4,784 targets mapped to the 3,200 and 4,218 Ensembl human genes with sequence identity 100% and greater than 90%, respectively. The 4,784 targets with sequence identity above 90% were used in our analysis, with 2,180 of them having GO or EC terms assigned.

Sequences were assigned GO terms from the EBI GOA resource (http://www.ebi.ac.uk/GOA). The query sequence was aligned with the UniProt GO annotated sequence with Blast [41]. If the Blast sequence identity was above 40%, and the overlap was above 90%, the annotated GO terms were mapped to the query gene (16,211 for GO molecular function, 14,876 for GO biological process, and 13,322 for GO cell component). The threshold is based on the observation that below 40% sequence identity with global alignment, the functional similarity relationship breaks down [10,15]. Sequences were also mapped to enzyme classification numbers with the annotations and sequences in the UniProt database as the reference. The 40% sequence identity and 90% overlap threshold was also applied to EC mapping.

Genome sequences were masked for low-complexity regions, coiled-coils, and transmembrane helical domains, using SEG [43], Coils [44], and TMHMM [45], respectively. SCOP superfamily domains [46] of unmasked regions of human genome sequences were assigned with HMMER [47]. A set of hidden Markov Models of SCOP domains was taken from SUPERFAMILY 1.65 [48]. Given the current stage of homology modeling, the model was usually reliable when the sequence identity was above 30% between the query sequence and the template structure [25]. Thus, only those assigned domains with sequence identity above 30% in the alignment were considered as homology models. The sequence regions that were not assigned by SCOP domains were further parsed with Pfam 16.0 [49]. The remaining unmasked sequence segments that were not annotated by either SCOP or Pfam but longer than 30 residues were considered as novel domains. Moreover, for contiguous domains, their orders were recorded in the database. The two domains were considered as contiguous with each other if they were not separated by the filtered sequence segments.

All genome sequences were clustered with 40% sequence identity and 90% overlap using CD-HIT [50].

For PDB structures and structural genomics targets, SCOP domains and their arrangements were computed with the same procedure as for genome sequences.

The original mapping of structures to OMIM numbers was taken from SWISS-PROT [51]. The mapping of genome sequences to OMIM numbers was from NCBI [52]. These mappings were recorded and used from the PDB beta site [26].

### Data analysis.

For each functional or structural category, the number of sequence or structure clusters in the subcategory was normalized with that of sequence clusters from the genome. The overall similarity between two distributions—for example, the PDB structure and the human genome—was measured with Kendall's tau correlation coefficient *τ* [53]. For N pairs of measurements (xi, yi), each of them has N(N−1)/2 pairs of data points. *τ* is computed as:


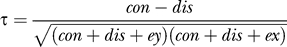


*con* is defined as the number of pairs where (xi, xj) ranks the same as (yi, yj). *dis* is the number of pairs where (xi, xj) ranks the opposite to (yi, yj). *ey* is the number of pairs where yi = yj, and *ex* is the number of pairs where xi = xj.

Kendall's tau correlation coefficient ranges from −1.0 to 1.0. If two measurements have the similar ordering, it will be close to 1.0. The opposite ordering will give values close to −1.0. The coverage was also computed and defined as the ratio between the number of functional categories that have at least one structure representative and all functional categories.

### Data access.

Data were warehoused in a single relational database where relations represent the mappings between the individual data sources. From a user's perspective, data appear in a multi-dimensional space. Each of the functional or structural categories is considered one dimension in the multi-dimensional space. A PDB structure or a genome sequence occupies a cube in this space. Any combination of two dimensions can be selected, and the distributions corresponding to the selected dimensions are calculated and displayed. The dimensions are organized in a hierarchal fashion according to their functional or structural taxonomies. Thus, data mining tasks such as drill-down or roll-up are supported. The database is accessible from http://function.rcsb.org:8080/pdb/function_distribution/index.html.

## Supporting Information

### Accession Numbers

The UniProt (http://www.pir.uniprot.org/) accession number for prostate specific kallikrein of serine proteases is P07288.

## Abbreviations

EC: Enzyme Commission
GO: Gene Ontology
OMIM: Online Mendelian Inheritance in Man
PDB: Protein Data Bank
